# Neutralization of ricin toxin on building interior surfaces using liquid decontaminants

**DOI:** 10.1371/journal.pone.0302967

**Published:** 2024-05-09

**Authors:** William R. Richter, Bailey L. Weston, Michelle M. Sunderman, Zach Willenberg, Katherine Ratliff, Joseph P. Wood

**Affiliations:** 1 Battelle Memorial Institute, Columbus, OH, United States of America; 2 U.S. Environmental Protection Agency, Office of Research and Development, Research Triangle Park, NC, United States of America; VIT University, INDIA

## Abstract

Ricin is a highly toxic protein, capable of inhibiting protein synthesis within cells, and is produced from the beans of the *Ricinus communis* (castor bean) plant. Numerous recent incidents involving ricin have occurred, many in the form of mailed letters resulting in both building and mail sorting facility contamination. The goal of this study was to assess the decontamination efficacy of several commercial off-the-shelf (COTS) cleaners and decontaminants (solutions of sodium hypochlorite [bleach], quaternary ammonium, sodium percarbonate, peracetic acid, and hydrogen peroxide) against a crude preparation of ricin toxin. The ricin was inoculated onto four common building materials (pine wood, drywall joint tape, countertop laminate, and industrial carpet), and the decontaminants were applied to the test coupons using a handheld sprayer. Decontamination efficacy was quantified using an *in-vitro* cytotoxicity assay to measure the quantity of bioactive ricin toxin extracted from test coupons as compared to the corresponding positive controls (not sprayed with decontaminant). Results showed that decontamination efficacy varied by decontaminant and substrate material, and that efficacy generally improved as the number of spray applications or contact time increased. The solutions of 0.45% peracetic acid and the 20,000-parts per million (ppm) sodium hypochlorite provided the overall best decontamination efficacy. The 0.45% peracetic acid solution achieved 97.8 to 99.8% reduction with a 30-min contact time.

## Introduction

Ricin is a highly toxic protein derived from the beans of the *Ricinus communis* (castor bean) plant and is capable of inhibiting protein synthesis within cells, ultimately resulting in cell death. *Ricinus communis* plants can be found at most local nurseries for use as an ornamental plant and agriculturally are harvested to produce castor bean oil. The median lethal dose (LD_50_) of ricin in mice is 5 micrograms per kilogram (μg/kg) via intravenous (IV) injection and 10–15 μg/kg for nonhuman primates via aerosol exposure [1]; extrapolation of these indicate human LD_50_ exposure could be ~1 to 5 mg/kg IV. The Department of Energy’s Temporary Emergency Exposure Limit for one hr of ricin exposure in the air is 8.2ng/m^3^ [2]. Ricin poisoning may occur through consumption of tainted food, direct injection, dermal or ocular contact, or via inhalation, and most likely resulting from ricin powder being placed into an article of mail [1]. A previous study demonstrated that minimal loss of ricin toxin bioactivity occurred on building material surfaces at 28 days and 20°C [3], highlighting the need to establish effective decontamination techniques to successfully remediate buildings or other critical infrastructure, to minimize ricin exposure to responders and building occupants.

Due to its high toxicity, readily available access to the plant, and ease of production, ricin has been used numerous times for nefarious purposes, since the 1978 assassination of Georgi Markov, which resulted from an injected pellet of ricin toxin disguised in the tip of an umbrella [4]. The U.S. government considered ricin toxin for use as a chemical warfare agent dating back to 1918 [5]. Since 2003, numerous letters containing ricin toxin have been sent through the mail to various locations, including the office of the New York City mayor, the Pentagon, and the White House [6,7]. Most recently in 2020, ricin was discovered in a package sent to former President Donald Trump [8]. The creation of the letters and packages containing ricin had the potential to contaminate the location where the ricin was produced as well as all subsequent pieces of postal processing equipment and associated infrastructure throughout the route of transit, creating exposure risks for the public as well as those handling the mail. Other ricin contamination events have occurred within the United States, prompting a coordinated response by the U.S. EPA with local communities to effectively remediate these affected areas [9]. While the quality of the ricin toxin used for these types of incidents is not publicly known, it is assumed that a crude preparation is more likely due to specialized equipment needed for purification of the toxin. Studies have also shown that a crude preparation of ricin toxin was generally more persistent on materials when directly compared to a purified form of ricin [3], and was therefore selected for this study.

While a few studies have examined the decontamination efficacy of liquid decontaminants or treatments against ricin toxin [10–14] these have been primarily focused on food or liquid-based contamination (*in vitro*). Additional research to assess ricin neutralization has been conducted with fumigants (gaseous treatments) such as hydrogen peroxide (HP) vapor and chlorine dioxide (ClO_2_) gas, which could be used in large complex buildings or spaces. One such study examined the effects of vapor phase HP and found that when a pure or crude form of ricin was exposed to 400 ppm vapor phase HP for 14 hr, greater than 99% reduction was achieved [15] on stainless steel, rubber, plastic, aluminum, industrial carpet, ceramic tile, concrete, and paper. Another study found that a 99% reduction of pure ricin was achieved using 1,500 ppm of ClO_2_ gas and a contact time of 20 min on glass, painted concrete, galvanized metal, laminate, particle board, and industrial carpet [13].

Fumigation of large buildings can be challenging requiring complex equipment and may generate large volumes of potentially hazardous vapors or gases. The use of commercially available decontaminants applied via a spray offers a potential simple solution which could be broadly implemented for a crude ricin preparation and helps to mitigate the safety challenges associated with the use of many fumigants.

The primary objective of this study was to evaluate the neutralization of a crude preparation of ricin toxin, inoculated onto common building materials (pine wood, drywall joint tape, countertop laminate, and industrial carpet) using several commercially available liquid decontaminants and/or household cleaners. The decontaminants selected included solutions comprised of sodium hypochlorite (SH; i.e., three concentrations of chlorine bleach), quaternary ammonium compounds (QAC), sodium percarbonate (SP), peracetic acid (PAA), and aqueous HP. The number of spray applications and contact times (the elapsed time the decontaminant was in contact with the coupon/ricin) were also test variables. Decontamination efficacy was quantified as the percent reduction in the mass of ricin toxin recovered from test coupons (sprayed with the decontaminant) compared to the mass of toxin recovered from positive control coupons (not sprayed with the decontaminant).

## Materials and methods

### Ricin toxin

Testing was conducted using two forms of ricin toxin throughout the study, a purified form and a crude form. The purified form was used to develop standards for the calibration curves used in the bioassay and was prepared for the study using a single lot of commercially prepared purified ricin toxin (Cat. No L-1090, Ricin communis agglutinin II, certified at 20 mg/mL protein concentration: Vector Laboratories, Burlingame, CA). It had been kept for long-term storage at ≤ -70°C. This commercially prepared purified ricin toxin was diluted to a concentration of 1,950 ng/mL and stored in sterile phosphate buffered saline (PBS; Cat SH30256.02 Cytiva HyClone, Marlborough, MA) at 2–8°C during the study. A second lot of purified ricin (from the same source) was required for use as a standard after degradation of the initial working stock pure ricin concentration was observed during testing. This second lot of purified ricin was also diluted to a concentration of 1,950 ng/mL and stored in sterile PBS at 2–8°C for the remainder of the study.

The crude preparation of ricin was inoculated onto test materials and used to assess decontamination efficacy. It was extracted from whole castor beans (Sheffield’s Seed Company, Locke, NY) and prepared in the laboratory using methods derived from scientific literature [16], consistent with a previous study [3]. A single lot of crude ricin extract was made and used throughout the study. Briefly, whole castor beans were de-husked and homogenized into a slurry, precipitated from the solution, and dialyzed with sterile PBS (Cat #D8537 Sigma-Aldrich, St. Louis, MO). The final crude ricin extract was prepared in sterile PBS and stored at 2–8°C. The initial concentration of this extract was assessed and found to be ~11.8 mg/mL, and then the concentration was determined each day of testing.

All tests were performed in a containment area designed to meet or exceed the biological safety level-3 facility guidelines published by the Centers for Disease Control and Prevention and the National Institutes of Health [1].

### Test materials and inoculation

**Fig 1:**
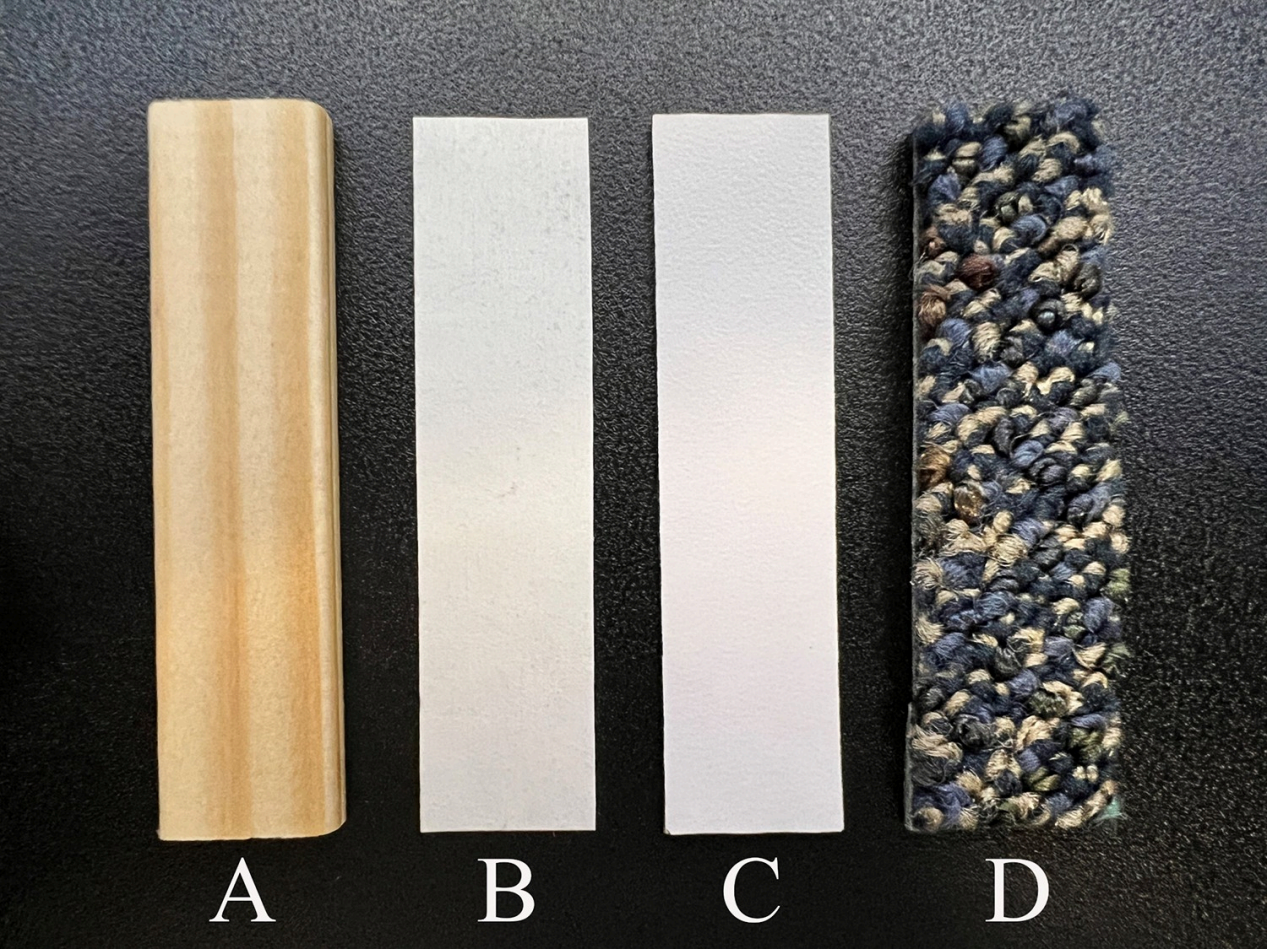
Coupon materials pine wood (A), joint tape (B), laminated countertop (C), industrial carpet (D).

Four common indoor building materials (Fig 1) that represented both porous and non-porous materials were selected for testing: pine wood (Item #3542, Lowe’s, Hilliard, OH), joint tape to represent drywall paper (Item #11749, Lowe’s), laminated countertop (Item #912842, Lowes, Hilliard, OH), and industrial carpet (Shaw Swizzle EcoWorks Style 10401, Shaw Floors, Dalton, GA). The test coupons (1.9 cm × 7.5 cm) were cut from larger pieces of representative stock material. Each test material was visually inspected for physical integrity prior to and after testing to assess any damage or change to the coupons. Coupons were sterilized to eliminate the presence of endogenous microorganisms, which could potentially interfere with the cell-based bioassay used for determining ricin bioactivity. Sterilization was performed by electron beam (E-beam) irradiation with a dose of ~200 kGy (E-beam Services Inc., Lebanon, OH) prior to testing.

The crude ricin toxin was inoculated onto the test materials to assess the neutralization efficacy of various liquid decontaminants as a function of material type and contact time (the time the decontaminant was in contact with the coupon/ricin). Positive controls (not exposed to the decontaminant; three replicates were used for each positive control material for each experiment) and test coupons were arranged on a flat rack in a Class II biological safety cabinet (BSC; Model SG603A, The Baker Company, Sanford, MN, USA) under ambient environmental conditions, ~22°C and 40% relative humidity (RH) during testing. Coupons were grouped by test material and then further separated between positive control and test sample. Each group contained one blank sample (negative control) with no crude ricin toxin applied and three sample coupons inoculated individually with crude ricin toxin.

The concentration of crude ricin toxin was determined each day of testing using a cell-based bioassay (discussed below). This concentration was multiplied by the volume of crude ricin inoculated onto each positive control and test sample coupon to determine the mass of crude ricin placed onto each coupon (the inoculum volume of 21.25 microliter [μl] was determined based on the initial titer of the crude ricin just after production, to achieve a target mass inoculum of 250 μg). For the study, this averaged (± SD) approximately 413 ± 156 μg per coupon. Dispensing of the inoculum was done via micropipette and applied in a single streak across the coupon. This technique allowed for decreased drying time and allowed for greater distribution across the coupon. Post inoculation, samples dried in the BSC for approximately 1 hr at ambient conditions, ~22°C and 40% RH.

### Decontaminants and application procedures

Twelve experiments were conducted for the study. The decontaminants evaluated included ready-to-use solutions of 3% aqueous HP (Meijer, Grand Rapids, MI) and a quaternary ammonium-based cleaner (Formula 409^®^ multi-surface cleaner, EPA Reg. #5813–73, Clorox Co., Oakland, CA). Additionally, three concentrations of SH (Clorox^®^ bleach, EPA Reg. #67619–32, 1,000, 7,500, and 20,000 ppm), a percarbonate solution (Oxiclean™ Laundry and Home Sanitizer, EPA Reg #10722–25, Church & Dwight Co., Ewing, NJ) and peracetic acid [PAA (Minncare^®^ Cold Sterilant; 4.5% PAA, EPA Reg. #52252–4, Minntech Corporation, Minneapolis, MN)] were prepared fresh each day these decontaminants were tested. SH was diluted using cell culture grade water (Corning, Cat #25-055-cm, Corning, NY) and evaluated using a Hach hypochlorite iodometric titration method (Hach, Method # 10100, Loveland, CO) to verify concentration of free available chlorine each day of testing. (The 20,000 ppm SH solution is essentially a one in four dilution of bleach containing at least 8% sodium hypochlorite.) The 0.45% PAA solution used in tests was prepared by performing a 1:10 dilution of the 4.5% PAA using cell culture grade water. SP was prepared by dissolving 71.17 g of powdered reagent into 1.0 liter of cell culture grade water. Refer to Table 1 for a summary of the decontaminants and test matrix. Each experiment (there were 12 experiments conducted for the study) utilized all four test materials, a unique decontaminant or decontaminant concentration, and either one or two contact times. With the exception of PAA, which was evaluated at contact times of 15, 30 and 60 min, all other decontaminants were evaluated at contact times of 30, 60 and 120 min. A contact time of 15 min was used in lieu of 120 min for the PAA, due to its high efficacy when initially tested at 30 min.

**Table 1:** Test matrix.

Liquid Decontaminant	EPA Reg. #	# Sprays	Contact Time (min)
SH 1,000 ppm	67619–32	5	30
SH 1,000 ppm	67619–32	5	60
SH 1,000 ppm	67619–32	10	120
SH 7,500 ppm	67619–32	5	30
SH 7,500 ppm	67619–32	5	60
SH 7,500 ppm	67619–32	10	120
SH 20,000 ppm	67619–32	5	30
SH 20,000 ppm	67619–32	5	60
SH 20,000 ppm	67619–32	10	120
SP	10772–25	5	30
SP	10772–25	5	60
SP	10772–25	10	120
QAC	5813–73	5	30
QAC	5813–73	5	60
QAC	5813–73	10	120
0.45% PAA	52252–4	5	15
0.45% PAA	52252–4	5	30
0.45% PAA	52252–4	5	60
3% HP	NA	5	30
3% HP	NA	5	60
3% HP	NA	10	120

SH = sodium hypochlorite (chlorine bleach); SP = sodium percarbonate; QAC = quaternary ammonium compound; PAA = peracetic acid; HP = hydrogen peroxide.

**Fig 2:**
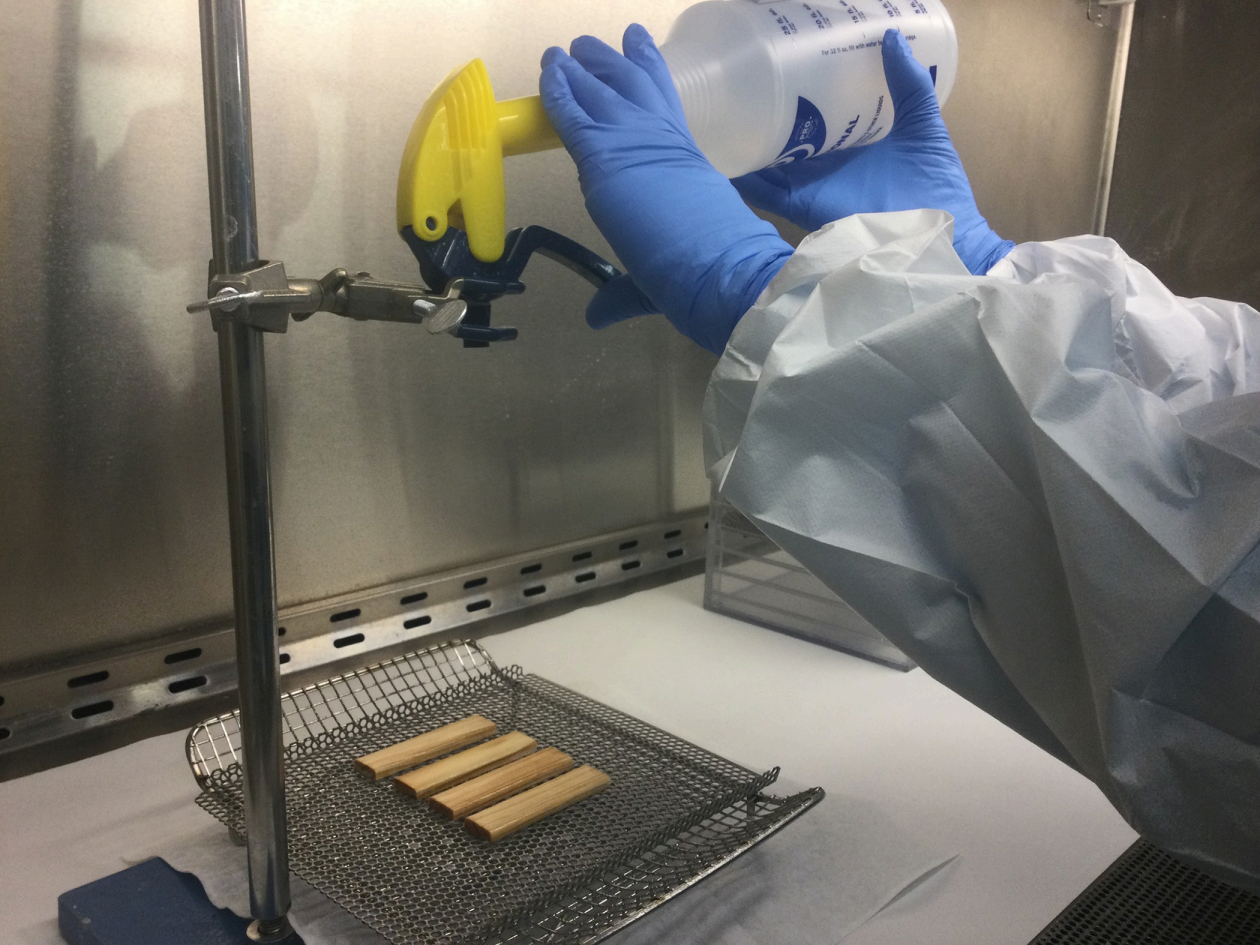
Spray application stand.

Once the inoculum on the test materials had dried (coupons were given a 1-hr dry time), a set of four coupons (three test replicates and one negative control) were transferred to the exposure application stand. Each group of test materials was placed atop a stainless-steel wire mesh tray in a horizontal position. The decontaminants were applied to the test materials using a handheld professional spray bottle (ZEP Inc., Emerson, GA) 20.3 cm above coupons (Fig 2). Each application of decontaminant consisted of five full sprays with the nozzle adjusted to the widest angle possible to ensure full coverage of liquid decontaminant across all four coupons in the grouping. One application (five sprays) of decontaminant was used for contact times of 15, 30, and 60 min. The contact time commenced (T = 0) once the coupons were initially sprayed. Two applications (10 sprays) were applied for the 120-min contact time, with the first application (five sprays) at T = 0 min and a second application (another five sprays) at T = 60 min. Once the liquid decontaminant had been applied, the rack containing the test materials was moved to a storage area within the BSC for the contact time allowing the next set of test coupons to be processed. All positive control samples (without decontaminant application) were held in the storage area within the BSC for the duration of the longest contact time.

### Sample processing and ricin toxin quantification

At each predetermined timepoint, each test coupon and associated blanks (negative control) were removed from the test storage area and placed into its own 50 mL conical tube containing 10 mL of complete growth media (Dulbecco’s Modified Eagle’s Medium, Gibco Cat. No. 10566016, Carlsbad, CA) supplemented with 10% fetal bovine serum (Gibco Cat. No. 10082147) and 1% penicillin-streptomycin (Gibco Cat. No. 15140122). Positive control coupons were placed into conical tubes as described above for the test coupons, but after conclusion of the longest contact time. The conical tubes were capped, placed on their sides, and agitated on an orbital shaker for 15 min at approximately 200 rpm at room temperature to extract any remaining ricin from the coupon. The presence of residual active toxin from the test and control coupon extracts was determined using the bioassay described below. The percent recovery of ricin from the positive controls was then determined based on the mass of ricin recovered from positive controls divided by the mass of ricin inoculated.

Ricin toxin exerts its toxic effect through inhibition of protein synthesis within the cell, which leads to cell death. Assays such as electrochemiluminescence (ECL) or enzyme-linked immunosorbent assay (ELISA) [17] can detect ricin toxin using antibodies and may be appropriate for environmental samples when the presence of cytotoxins besides ricin is not known. In this laboratory study, a cytotoxicity assay was used, since cytotoxic effects other than ricin, such as potentially from the materials or decontaminants used, could be controlled. Therefore, an *in vitro* cytotoxicity assay was used to quantitate the level of bioactive ricin toxin extracted from both the decontaminated and positive control material coupons. The bioassay used in this evaluation for determining the cytotoxicity of bioactive ricin toxin is based on the 3-(4,5-dimethylthiazol-2-yl)-2,5-diphenyltetrazolium bromide (MTT) assay [16]. The use of the MTT assay allowed us to directly measure and quantify the toxicity of the ricin, whereas the ELISA method only confirms the presence of ricin protein structures and does directly measure whether the ricin is still toxic following decontamination. Thus, ELISA may detect inactivated toxins. To determine the concentration of ricin toxin from each test sample, a ricin standard (using the pure form described above) was assayed in parallel on each test plate. Dilutions of the ricin standard were used to prepare a seven point-standard curve of absorbance versus calculated mass of ricin toxin protein. This methodology has been described in further detail previously [3].

Cytotoxicity, reported as mass of bioactive toxin, was determined using a reference standard prepared from the purified form of ricin toxin. Initially, and then throughout the study, the potential inherent cytotoxicity of material coupon extracts as well as residual decontaminant chemistry was assessed to mitigate any potential confounding cytotoxic effects observed in the assay. To account for this potential bias, the dilution factor of coupon extracts exhibiting less than 20% cytotoxicity when compared to negative controls (cell culture media only) was used as the starting dilution for each corresponding test material, decontaminant, and contact time. When applicable, results from this variable starting dilution were then used as the limit of detection for that sample. The growth media used for extraction of ricin from the coupons provided sufficient quenching for all the decontaminants’ active ingredients except for the QAC multi-surface cleaner. For this decontaminant, the addition of 1.77 g of sodium thiosulfate to each extraction buffer tube was required to neutralize any remaining QAC from the decontaminant.

### Data analysis

The percent reduction of ricin toxin was assessed by determining the mass of bioactive ricin extracted from each test coupon post-decontamination as compared to the average mass of bioactive ricin extracted from the corresponding positive control coupons.

(1)Decontamination of ricin in terms of percent reduction for a given test condition (decontaminant, time point, and material) was calculated as the difference between the mean positive control mass values and the mean test mass values, divided by the mean control mass values, i.e.:

$$percent\;reduction=\frac{{\bar{Massc}}_{ij}-{\bar{Masst}}_{ij}}{{\bar{Massc}}_{ij}}*100\%=\left(1-\frac{{\bar{Masst}}_{ij}}{{\bar{Massc}}_{ij}}\right)*100\%.$$ (1)

where Massc_*ij*_ refers to the *j* individual mass values for material “*i*” obtained from the positive control coupons, Masst_*ij*_ refers to the *j* individual mass values for material “*i*” obtained from the corresponding test coupons, and the overbar designates a mean value. In this study, there were three positive controls and three corresponding test coupons (*i*.*e*., *j* = 3) for each coupon material “*i”* for each test condition and each contact time.

### Statistical analysis

(2)For the purposes of conducting statistical analysis, the percent recovery of ricin was calculated as the ratio of log-transformed mean test coupon ricin mass values to log-transformed mean control mass values, i.e.:

$$percent\;receovery=\frac{{log}_{10}({\bar{Masst}}_{ij})}{{log}_{10}({\bar{Massc}}_{ij})}$$ (2)

where Massc_*ij*_ refers to the *j* individual mass values obtained from the positive control coupons, Masst_*ij*_ refers to the *j* individual mass values obtained from the corresponding test coupons, and the overbar designates a mean value. In this method, a smaller ratio is associated with a larger reduction. The assumption of normality for the dataset was more reasonable for the log-transformed recovery values than the untransformed values and allowed better detection of statistically significant differences between sample means. Thus, all models were fitted to the log-transformed values prior to analysis, consistent with a previous ricin attenuation study [3] (Wood et al., 2018). In Eq 2, each individual test sample percent log recovery was calculated, allowing better modeling power with more data points (three points per condition versus one average). Since the individual control recoveries do not necessarily correspond to a specific test sample, calculating percent recovery using one arbitrary control sample of the three tested may not accurately reflect decontamination activity, so the average of the three is used.

Analysis of variance (ANOVA) models were fitted to the log-transformed ratios calculated from Eq 2. Models included main effects for material and decontaminant application (factor of contact time and total number of sprays applied), and the interaction of material and application. Pairwise comparisons were performed to test for significant differences between each combination of application and material. Both unadjusted and Tukey-adjusted p-values were determined; for the purpose of this analysis, the effects of the test variables are reported as significant if the Tukey-adjusted p-values were ≤ 0.05. All statistical analyses were performed using R [18,19].

## Results

**Fig 3:**
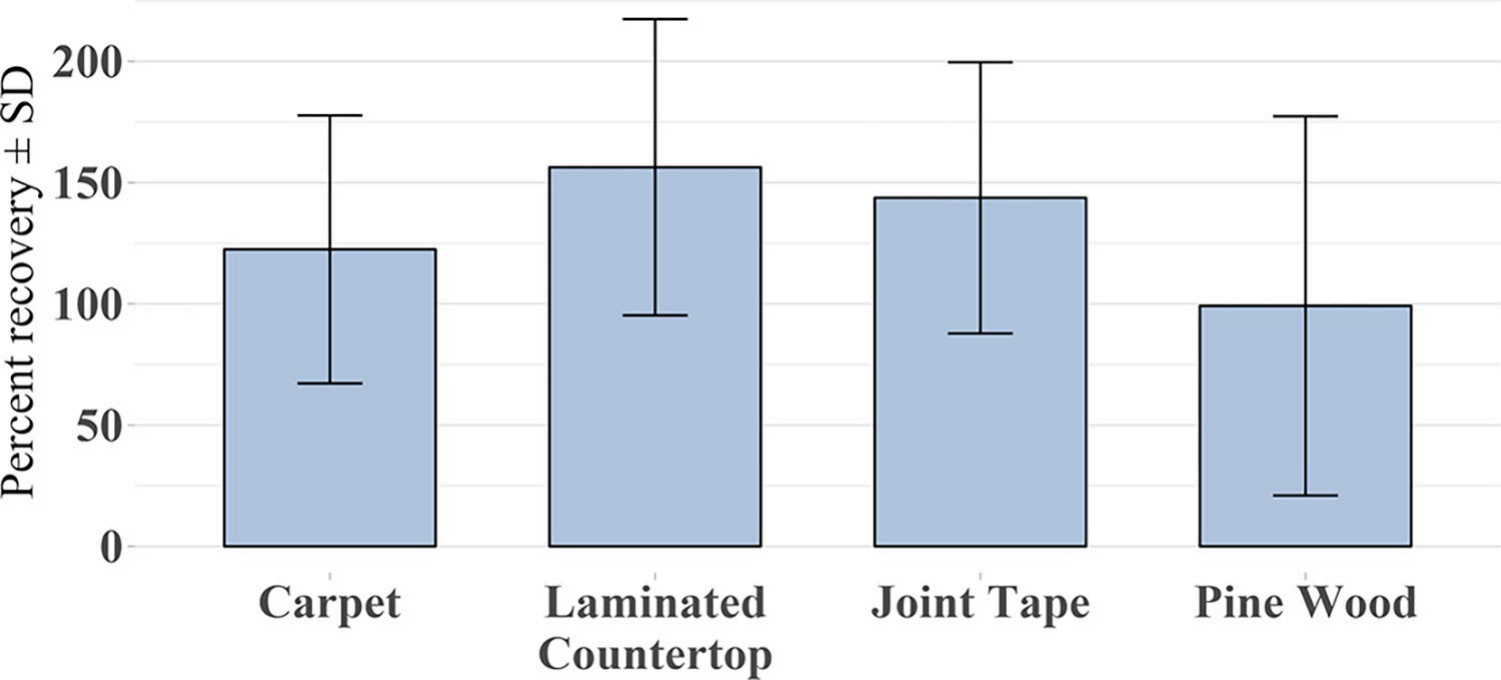
Study-wide average percent recovery of ricin ± SD from positive control coupons by material (n = 36 for each material).

The study-wide average percent recoveries of ricin from the positive controls for each of the four materials (n = 36 for each material; 3 replicate positive controls per experiment × 12 experiments) are shown in Fig 3 and ranged from 99% (pine wood) to 156% (laminated countertop). These are the study-wide averages of the percent ricin recovered relative to the quantity of ricin inoculated onto the coupons. The percent recoveries were relatively variable as noted by the standard deviation error bars.

**Fig 4:**
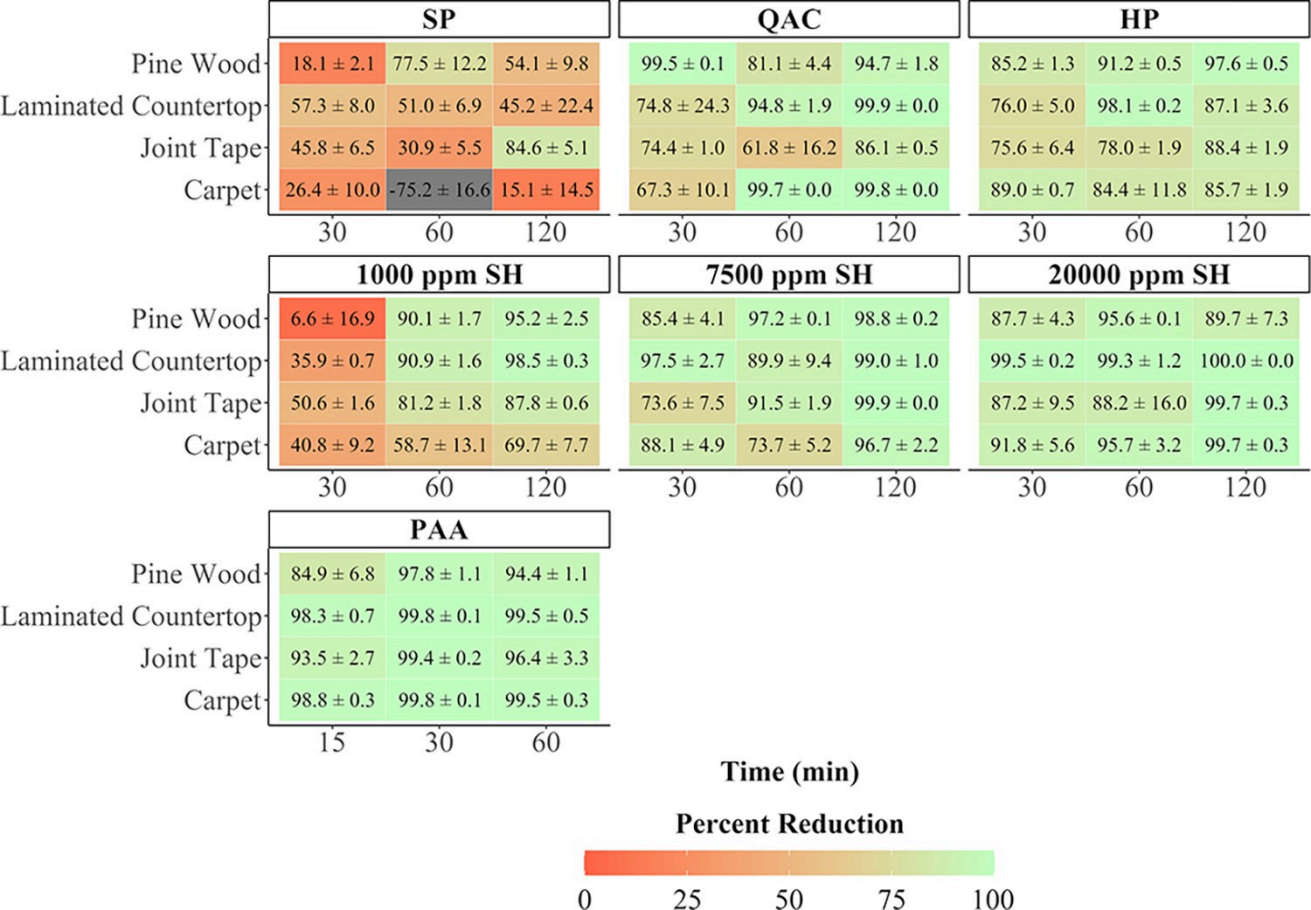
Average percent reduction of ricin ± SD for each decontaminant, by material and application contact time.

The average percent reductions of ricin toxicity by material, decontaminant, and contact time are shown in Fig 4. Overall, the average percent reductions ranged from 6.61% to 99.97%, excluding one outlier (SP on carpet for 60 min) which resulted in -75.15% reduction. This negative efficacy was the result of an irregularly low recovery from the positive controls during that experiment (if the efficacy for this test point was calculated using the average positive control recovery for carpet across all tests, the adjusted percent reduction would have been 24.42%). The 0.45% PAA solution was the most effective decontaminant, achieving the highest percent reduction of ricin for the 30 min/5× spray application on all materials, as well as the highest average percent reduction across all materials and contact times tested. Even at the least favorable application conditions, the minimum efficacy the 0.45% PAA achieved was 85%. The SP-based laundry additive and cleaner was the least effective. The decontamination efficacy for the three SH solutions improved with increasing concentration of the hypochlorite, with the 20,000 ppm SH solution performing the best. This decontaminant provided over 90% efficacy for eight of 12 of the material/application conditions tested. Excluding the least effective decontaminants (SP and 1000 ppm SH), the lowest efficacy achieved overall with the 30-min contact time was 67% using the QAC decontaminant.

**Fig 5:**
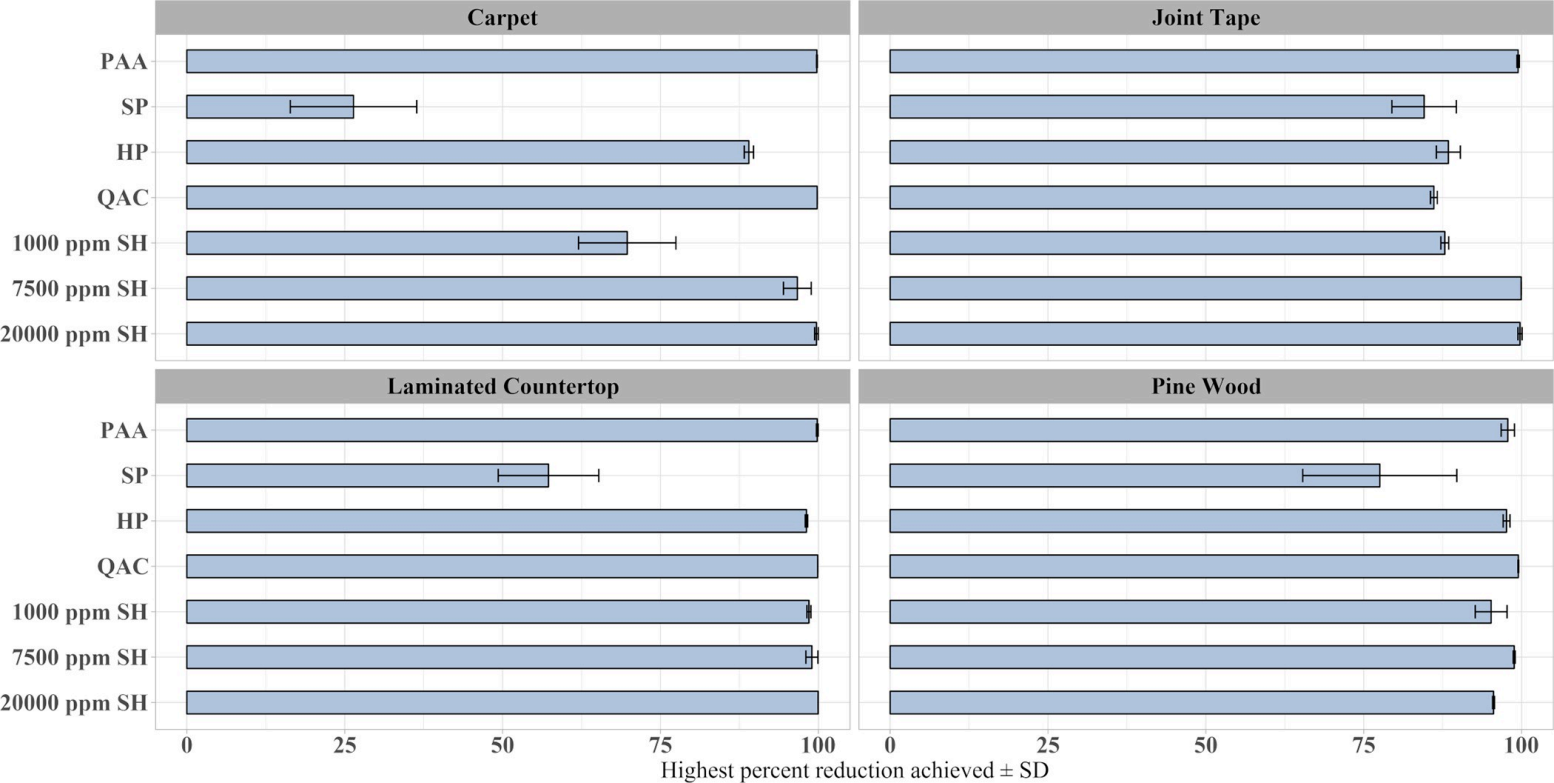
Highest percent reduction achieved by each decontaminant for each material ± CI.

Overall, 15 cases out of 84 combinations of decontaminant, application, and coupon material resulted in ≥ 99% reduction of ricin toxin. As seen in Table 2, the decontaminants responsible for most of the instances where ≥ 99% reduction of ricin was achieved were PAA, the QAC, and 20,000 ppm SH. The PAA formulation achieved ≥ 99% reduction with 30- and 60-min contact times, while the SH and QAC mostly required 120 min to achieve ≥ 99% reduction. As an alternative visualization of the data, the highest mean decontamination efficacy achieved by each decontaminant for each material type is shown in Fig 5.

**Table 2:** Testing parameters resulting in ≥ 99% mean percent reduction.

Decontaminant	Decontaminant Application		Material	Mean Percent Reduction
	Contact Time (min)	Total Sprays		
7,500 ppm SH	120	10	Joint tape	99.93%
20,000 ppm SH	30	5	Laminated countertop	99.47%
	60	5	Laminated countertop	99.27%
	120	10	Laminated countertop	99.97%
	120	10	Carpet	99.71%
	120	10	Joint tape	99.74%
QAC	30	5	Pine wood	99.48%
	60	5	Carpet	99.69%
	120	10	Carpet	99.81%
	120	10	Laminated countertop	99.90%
0.45% PAA	30	5	Carpet	99.77%
	30	5	Laminated countertop	99.83%
	30	5	Joint tape	99.44%
	60	5	Carpet	99.47%
	60	5	Laminated countertop	99.45%

The decontaminants’ efficacy generally improved (although not in every case) as the number of spray applications or contact time increased. However, this effect was less pronounced with the PAA decontaminant, since it was already relatively effective with just a 15-min contact time. Laminated countertop was the most common material in which ≥ 99% reduction of ricin was achieved (making up 54.5% of instances), followed by carpet with 33.3% of instances. In the statistical analysis, material type was a significant main effect at the 0.05 confidence level of the p-value for all decontaminants. Of the pairwise comparisons for material type for each decontaminant tested, 28 of the 42 comparisons evaluated (66.7%) were statistically significant. The ranking of materials by the number of comparisons in which ricin toxicity was reduced to a significantly greater extent than the material it was compared with (shown in parentheses) is as follows: laminated countertop (12), pine wood (7), carpet (5), and joint tape (4).

Decontaminant application (contact time or contact time along with increased number of sprays) was also a significant main effect for all the statistical models. For 1,000 ppm SH, all pairwise comparisons of decontaminant application were statistically significant, with more robust applications (longer contact time and increased number of total sprays) resulting in a significantly greater percent reduction for each comparison. The decontaminants SP, QAC, 7,500 ppm SH, and 20,000 ppm SH had significantly greater percent reduction in ricin for the 120 min/10× spray application when compared to the two less robust applications tested. The 3% HP solution was the only decontaminant to undergo testing out to 120 min contact time where the 120 min/10× spray application did not result in a statistically greater percent reduction.

## Discussion

The spray application of liquid decontaminants is commonly used for routine disinfection in public health settings and was a common disinfection approach during the most recent COVID-19 pandemic [20–22]. Spray based decontaminants have also been evaluated for use against other select agents such as *Bacillus anthracis* on common interior materials [23,24]. The use of commercial off-the-shelf (COTS) decontaminants applied via readily available spray equipment can provide a broadly available and less expensive approach for decontamination of materials contaminated with ricin.

Previous studies have evaluated the efficacy of liquid decontaminants to neutralize ricin, but many of those focused only on ricin in a liquid solution (i.e., no material substrate was involved in the study) [10–12]. Studies have shown that material has a strong impact on decontamination efficacy, and that decontaminants are typically more effective in a liquid suspension without a material, compared to using a more realistic application approach such as spraying [25]. Two other studies for ricin neutralization investigated the efficacy of using gaseous or vaporous decontamination technologies [13,15]. Tolleson et al. [14] investigated the neutralization of pure ricin inoculated onto stainless steel surfaces (with and without food residue) using liquid decontaminants, but these tests were conducted via immersion. While these studies provide needed efficacy data for ricin neutralization, this present study is novel in several ways: it evaluated decontamination efficacy using the more realistic and challenging crude ricin form; tests were conducted on common interior building materials (rather than on common laboratory substrates or in suspension); tests were conducted using readily available COTS cleaners and disinfectants; and were delivered as a spray, as would be done in a real incident.

In an actual ricin contamination incident, it is important to understand that the operational parameters and method of decontaminant application can influence efficacy. Choosing test parameters that provide the most realistic representation of use in the field is an important factor when designing these types of decontamination studies. This study focused on the decontamination of four types of common building materials (pine wood, joint tape, laminated countertop, and carpet). Decontamination tests were performed using a handheld COTS spray bottle delivering the selected decontaminant (SH, QAC, SP, PAA, and HP) on the surface of the test material to assess the neutralization of crude ricin toxin as a function of the number of spray applications and contact time.

The average percent recovery of ricin from the positive controls over the study ranged from 99% (pine wood) to 156% (countertop laminate) and is consistent with previous efforts except for pine wood [3]. The cause of the percent recovery being greater than 100% is unknown but is most likely related to the high variability of the crude ricin recoveries. This variability in positive control recovery in turn could be due to the large dilutions required of the crude ricin stock (for the cytotoxicity assay) when evaluating its ricin concentration each day of testing. Variability in recovery of ricin from the test materials may also be attributed to the use of a biological system for quantitation (a cell-based assay), as well as potential variability of ricin toxin associated with using actual castor beans, which may include additional, extraneous proteins or other constituents in the crude suspension. Some of the variability in ricin recovery may also be due to the use of realistic, porous building materials. While the study-wide percent recovery values were higher than 100% in most cases, it does not impact ricin neutralization efficacy calculations since the percent reduction on test coupons was determined relative to the recovery of ricin from the positive controls from each test. Ricin recoveries for a given material in a specific experiment were generally more precise than the study-wide averages.

While no conditions resulted in complete neutralization of the crude ricin toxin on all test materials (i.e., there were no cases in which ricin was not detected following decontamination), 15 cases out of 84 combinations of decontaminant, application, and coupon material resulted in ≥ 99% reduction of ricin toxin. PAA, QAC, and two bleach solutions (7,500 ppm and 20,000 ppm SH) exhibited the best performance overall at the longest contact durations of 60 and 120 min, respectively. The 0.45% PAA was the overall best performer and achieved 94.4 to 99.5% reduction with a 60-min contact time. This is consistent with other decontamination studies, in which PAA at a similar concentration has also been shown to be a very effective decontaminant for other threat agents such as *Bacillus anthracis* [26,27]. The 20,000-ppm bleach solution was also relatively effective for ricin neutralization with the 2-hr contact time and is also consistent with a study evaluating bleach at that concentration for inactivation of *Bacillus* spores on outdoor materials [28]. The 20,000-ppm bleach solution can be made using a one in four dilution of bleach containing at least 8% sodium hypochlorite (the approximate starting concentration used in this study).

Statistical analysis confirmed that the decontaminant application approach (i.e., the number of sprays coupled with the contact time) was a main effect, that is, increasing the total number of sprays or increasing the contact time significantly increased the efficacy of the selected decontaminant. This was true for samples that received five sprays and observed contact times within the first 60 min of application, as well as those that were held out to 120 min and received an additional five sprays to maintain wetness on the coupon surface. Further research is needed to confirm if additional spray applications and added contact time would result in further neutralization of ricin below detectable limits.

Statistical analysis also showed that material type was a significant factor in the selected decontaminants ability to neutralize ricin, consistent with other decontamination studies.

## Conclusions

While several of the decontaminants provided over 90% reduction of ricin, these were primarily achieved with the 10-spray, 2-hr contact time application. The 0.45% PAA solution provided over 97% efficacy for the four materials in just 30 min, and the 20,000 ppm SH solution provided over 89% efficacy for the four materials in 120 min. Overall, there were no test conditions in which ricin levels were reduced to below detection limits. To judge whether a 90% or a 99% reduction in ricin would be considered an appropriate goal for a decontaminant’s efficacy, further research is recommended to assess the exposure from ricin that may remain on these materials following decontamination, and relatedly, determine the levels of ricin on building materials in which there would be no risk of health effects.
